# Preventive Measures of Middle Turbinate Lateralization After Endoscopic Sinus Surgery: An Updated Review

**DOI:** 10.7759/cureus.15763

**Published:** 2021-06-19

**Authors:** Rahmah A Alaryani, Riyadh A Alhedaithy

**Affiliations:** 1 Otolaryngology, Prince Sultan Military Medical City, Riyadh, SAU; 2 Otolaryngology, Ministry of National Guard Health Affairs, Riyadh, SAU

**Keywords:** middle turbinate, lateralization, medialization, synechia, prevention

## Abstract

Introduction: The middle turbinate (MT) is the most important anatomical structure inside the nasal cavity and a landmark in the identification of skull base, ethmoid cells, and lamina papyracea. Postoperatively, lateralizationof the MT can cause synechia and obstruction of the middle meatus and the maxillary, ethmoid, or frontal sinuses.

Objective: To review the current literature about the outcome of different techniques used intraoperatively to prevent lateralization of MT after functional endoscopic sinus surgery (FESS).

Materials and methods: This retrospective narrative literature review provides a summary of current and past research publications about different techniques used intraoperatively to prevent MT lateralization after FESS.

Results: Many methods have been described to prevent the lateralization of MT and synechiae formation. These methods include controlled synechiae, suture lateralization, metal clips, partial resection of MT, middle meatus implants, and steroid-eluting implants and stents.

Conclusion: The ideal FESS should include preservation of the MT, reducing its lateralization, and preventing synechia formation.Different techniques have been discovered in an attempt to prevent this complication.

## Introduction and background

The middle turbinate (MT) is an anatomical structure inside the nasal cavity that plays an important function in nasal air flow and olfaction [1]. It is considered a landmark in functional endoscopic sinus surgery (FESS) that helps in the identification of the skull base, ethmoid cells, and lamina papyracea [2-3]. Postoperatively, lateralization of MT may cause significant scarring and obstruction of the middle meatus, increasing the risk of recurrences and complications [4]. The position of the MT in relation to the ostiomeatal complex (OMC) and the sphenoethmoidal recess has been found to affect the drainage of all paranasal sinuses. For that reason, the interventions on MT during surgery might be required to achieve a better surgical outcome. However, floppy MT could happen mainly due to excessive excision of the basal lamella of MT, removal of the horizontal bony portion of MT, lateral fracture of MT to access sphenoethmoidal recess, or from trimming of severe polyposis of MT with a microdebrider. One of the most common complications of FESS is synechia formation due to instability and lateralization of MT toward the lateral nasal wall [5]. This will lead to OMC obstruction and increase the risk of disease recurrence and complications necessitating a revision surgery [6]. In this narrative review article, the outcome of different techniques used intraoperatively to prevent MT lateralization after FESS will be discussed.

## Review

Methods and materials

This retrospective narrative review covers literature from the period 1992-2019. An electronic search was done using PubMed, Medline, and Ovid Science Direct databases using the keywords “synechia, Middle Turbinates, Sinus surgery”. The search unearthed a large volume of diverse materials that have been used to prevent lateralization of MT after FESS. We included papers and texts published in English that met the objectives of the review.

Inclusion criteria

Patients who underwent FESS with varying intraoperative techniques to prevent the lateralization of the MT.

Exclusion criteria

Patients who underwent FESS without any known technique to prevent the lateralization of the MT.

Results and discussion

Anatomic Characteristics of MT

It is important to review the MT anatomy to understand the dynamics of MT lateralization. It has four different parts, an anterior buttress, a posterior buttress, a vertical attachment, and a horizontal attachment. The anterior buttress is a point of attachment of the MT to the lateral nasal wall below the agger nasi. The posterior buttress is a point of attachment to the lateral nasal wall near the posterior end of the MT. The vertical lamella attaches superiorly to the lateral cribriform plate lamella, providing stability for the anterior MT and makes the boundary between the cribriform plate and the ethmoid roof. The horizontal lamella (also called the ground lamella or basal lamella) is attached laterally to the lamina papyracea and extends downwards to the posterior buttress [7].

Incidence of MT Lateralization After FESS

Synechia formation between the MT and the lateral nasal wall is the most common surgical complication of FESS, which occurs in 43% of patients [8]. Other studies showed the rate of synechiae formation ranges between 1%-10% [9-10]. It may occur despite careful surgical technique and aggressive post-operative care [11]. Also, it was recently reported the incidence of synechia formation of approximately 15%-22% in a cohort study [12]. Instability and lateralization of the MT towards the lateral nasal wall is the most common significant factor (ranging from 22%-75%) requiring revision FESS [13-14].

Causes of MT Lateralization

One of the first steps of FESS is the medialization of MT to provide proper exposure of the OMC components. The MT might be destabilized after medialization, which can lead to its lateralization and formation of synechia when the raw mucosal surfaces are left in apposition [15-16]. MT lateralization will result in OMC obstruction leading to disturbance of mucociliary clearance and increased risk of disease recurrence [16]. No association has been discovered between MT lateralization and specific surgical interventions (septoplasty and concha bullosa reduction), or preoperative variables (sex, asthma, polyp status, primary vs. revision surgery) [12]. Formation of synechiae may become increased due to use of hemostatic materials to control bleeding, medialization of the MT, violation of the MT support as in resection of the basal lamella, presence of a concha bullosa or a large MT in close proximity to the lateral nasal wall, excessive removal of nasal mucosa, and inadequate debridement during the postoperative period [16-17].

The Debate of MT

MT is a key structure in FESS but the adequacy of its management remains controversial [18]. Some authors suggested that MT should be resected to avoid complications of its lateralization. However, other authors, prefer to preserve this structure due to its anatomic importance as a surgical landmark in revision FESS, and due to its functional importance in nasal air flow and olfaction [19].

Techniques to Prevent MT Lateralization After FESS

Many techniques have been designed to prevent synechiae formation [6-20]. Each technique offers various features, success rates and benefits to serve the purpose better. However, no single technique has been documented to be definitively superior to another; all have advantages and disadvantages [6]. Many absorbable and nonabsorbable materials have been used for the medialization the MT [21-22]. These techniques include controlled synechiae, suture lateralization, metal clips, partial resection of MT, middle meatus implants and steroid-eluting implants and stents.

1- Controlled synechiae (Bolgerization): This was developed by Bolger et al. in 1999, and has been found to be effective in the prevention of MT lateralization in 88% of patients [17]. It involves abrading the mucosa of the medial surface of the anteroinferior portion of MT and the mucosa of the nasal septum on the opposite side. This is done using a sickle knife or a microdebrider which results in permanent adhesions between the MT and the septum. This requires nasal packing to remain in place in the middle meatus for ten days to facilitate adherence of the MT to the nasal septum. However, this method may compromise airflow and olfaction [23-24]. Friedman and Schalch reported a modified approach using the microdebrider to create the mucosal abrasion on the septum and MT, followed by application BioGlue (bovine serum albumin and glutaraldehyde) (CryoLife Europa Ltd, Hampshire, United Kingdom) between the MT and septum. Then, A temporary nasal packing was then inserted intra-operatively for 3 minutes to press the MT and septum together then removed without the need for postoperative packing. This will avoid the unwanted effect on olfaction [25].

2- Conchopexy technique: The needle is introduced into the left nasal cavity and passed through the head of the MT, septum and the contralateral MT in a single pass then brought back to the right MT and passed through the septum to the initial side to be knotted with left MT.

Many authors reported endoscopic conchopexy technique as the most effective method to prevent MT lateralization with high success rate (90%-92%) [23]. It consists of fixation of MT to nasal septum using a 4-0 absorbable suture material [e.g., Vicryl (Ethicon, Somerville, NJ, USA), Polysorb (Coridien, Mansfield, MA, USA)] with the small round needle which introduced into the left nasal cavity and passed through the head of the MT, septum and the contralateral MT in a single pass then brought back to the right MT and passed through the septum to the initial side to be knotted with left MT [12]. This technique is more comfortable for patients because it needs no postoperative nasal packing removal and it does not impair olfaction [18-23]. However, it is considered a difficult to perform this technique in some situations specially to pass a needle through the solid bone of MT or through the narrow posterior nasal cavity when a septoplasty was not yet performed [26]. Also, this method may compromise airflow and olfaction [24]. For that reason, conchopexy can be more useful in patients post FESS and septoplasty and having unstable MT more than a bolgerization technique which can produce major injury to MT and a septal perforation [18]. A new technique described by Hudson S and Orlandi R which simplified the process of suturing to avoid difficult knot-tying within the nasal cavity [18]. Chen et al. reported that MT suture conchopexy did not lead to a significant reduction in rate of MT lateralization [12], but Lee MR et al., in 2011, reported that conchopexy created a significant improvement regarding the intra-operative access facilitation into middle meatus and significant improvement of post-operative outcomes [27].

3- MT Medialization with metallic clips: Medialization of MT using metallic clips is an effective method of preventing MT lateralization with success rates reaching up to 96% [28]. It consists of crushing and medializing the MT against the nasal septum bilaterally. A 1-cm incision was made in the septal mucosa facing the head of the MT on each side and mucoperichondral flap was elevated for a short distance and two surgiclips were used to fix the head of the MT to the septal mucopericchondral flap. This method was found to be an adequate and simple procedure, with its low rate of complications. Also, it will not increase the incidence of the nasal infection or affect olfaction but there is a risk of extrusion or aspiration of the clip. Metallic clip is not recommended in case of polypoid MT or previous history of submucosal resection of turbinate or missing septal cartilage to avoid iatrogenic septal perforation [4-28].

4- Partial resection of MT: The resection of the lower third of the MT that corresponds to the ethmoidal infundibulum by utilization of curved cutting scissors, or shaver is another method [6]. Partial resection of MT is an effective method of preventing MT lateralization with a success rate of 97% [29]. This procedure does not completely eliminate the possibility of a synechia formation after surgery [2]. The preservation of the superior aspect of the MT may cause lateralization and iatrogenic frontal sinus obliteration [30]. Postoperatively, this technique may significantly increase the incidence of recurrent sinusitis and prolong the healing period. It may also alter the normal physiologic structure of the MT especially when it is totally resected [5-6].

5- Middle meatus implants (silastic stent): Middle meatus implant is a technique proposed to place a proper size of inverted U-shape polymeric silastic sheet according to the size of the nasal cavity, the size of the MT, and the height and depth of the middle meatus. It is inserted between the middle meatus and caudal portion of the nasal septum and fixed it with mattress suture. The silastic sheet can be removed 10 to 14 days after FESS [6-30]. The size of the silastic sheet plays an important role in the outcome of this technique. If the height of the silastic sheet is too tall, it may cause mucosal injury and bleeding due to irritation of MT, while if it is too short may be displaced or extruded from the middle meatus. Fixation of the silastic sheet with ethmoid packing over a prolonged period might increase the risk of infection or fatal toxic shock syndrome [30].

Another technique described by Jebeles and Hicks is the application of a 1 cm × 1 cm Merocel pack (Medtronic Xomed, Jacksonville, Florida, USA) between the bulla ethmoidalis and MT, this pack is placed for two days to stabilize the MT [31]. However, this technique is not preferred for two reasons: (1) There is a high incidence of lateralization of MT and reobstruction of the middle meatus postoperative. (2) The symptoms related to nasal packing (severe pain, severe headache, nasal obstruction with breathing difficulties, persistent rhinorrhea, and recurrent bleeding) [6-32]. Gaskins reported a synechiae rate of 10.0% for the unpacked sinuses and 6.7% for the packed sinuses (various packing materials were used) [33], while Shikani found rate of 18% for the unpacked sinuses and 4% for the packed sinuses [11].

6- Steroid-eluting implants and stents: Bioabsorbable steroid-eluting sinus stents are inserted into the sinuses to maintain sinus patency, stabilize the MT, reduce synechia formation, reduce the need for systemic anti-inflammation medication, reduce medical and surgical interventions and improve outcomes. The slow release of steroids for a period of 30 days aims to reduce inflammation and edema of sinuses mucosa and improve wound healing [34-35]. Currently, there are two steroid-eluting (mometasone furoate) stents, including the PROPEL implants (Intersect ENT, Palo Alto, CA) and the BISORB Drug-Eluting Sinus Biopolymer Stent System. In August 2011, the US Food and Drug Administration (FDA) approved the first steroid-eluting sinus stent (PROPEL implants or Intersect ENT) for use in the ethmoid sinus surgery. In 2016-2017, the US Food and Drug Administration (FDA) approved it for use in surgery of the frontal sinuses and maxillary sinuses, respectively [36-37]. The BISORB drug eluting sinus biopolymer stent system that are approved by the China Food and Drug Administration (CFDA) and designed for placement in the ethmoid or frontal sinuses after FESS [34].

## Conclusions

The MT is an important surgical landmark during FESS. Many methods have been proposed in the literature as useful, effective measures for preventing MT lateralization and synechia formation after surgery. Most of those methods achieved a favorable success rate in preventing unstable, floppy MT with few difficulties and complications.
